# The association between antibodies to neurotropic pathogens and bipolar disorder: A study in the Dutch Bipolar (DB) Cohort and meta-analysis

**DOI:** 10.1038/s41398-019-0636-x

**Published:** 2019-11-20

**Authors:** Gijsje J. L. J. Snijders, Hans C. van Mierlo, Marco P. Boks, Marieke J. H. Begemann, Arjen L. Sutterland, Manja Litjens, Roel A. Ophoff, René S. Kahn, Lot D. de Witte

**Affiliations:** 1 Department of Psychiatry, Brain Center Rudolf Magnus, University Medical Center Utrecht, Utrecht, The Netherlands; 2 Department of Psychiatry, Academic Medical Centre (AMC), Amsterdam, The Netherlands; 3 Department of Translational Neuroscience, Brain Center Rudolf Magnus, University Medical Center Utrecht, Utrecht, The Netherlands; 4 Center for Neurobehavioral Genetics, University of California Los Angeles, Los Angeles, CA USA; 5 Department of Psychiatry, Icahn School of Medicine at Mount Sinai, New York, NY USA

**Keywords:** Physiology, Scientific community, Bipolar disorder

## Abstract

Exposure to neurotropic pathogens has been hypothesized to be a risk factor for the development of bipolar disorder (BD). However, evidence so far is inconsistent. We, therefore, analyzed the seroprevalence and titer levels of IgG antibodies against several herpesviruses and Toxoplasma gondii *(T. gondii)* in plasma of 760 patients with a bipolar disorder, 144 first-degree matched relatives and 132 controls of the Dutch Bipolar (DB) Cohort using ELISA. In addition, we performed a literature-based meta-analysis on the seroprevalence of IgG antibodies against these pathogens (*n* = 14). Our results in the DB Cohort and subsequent meta-analysis (*n* = 2364 BD patients, *n* = 5101 controls) show no association between exposure to herpesviruses and bipolar disorder (HSV-1 [adjusted OR 0.842, 95% CI 0.567–1.230], HSV-2 [adjusted OR 0.877, 95% CI 0.437–1.761], CMV [adjusted OR 0.884 95% CI 0.603–1.295], EBV [adjusted OR 0.968 95% CI 0.658–1.423]). In the DB Cohort, we did not find an association between bipolar disorder and *T. gondii* titer or seroprevalence either [adjusted OR 1.018, 95% CI 0.672–1.542]. The overall OR was not significant for *T. gondii* [OR: 1.4, 95% CI 0.95–1.90, *p* = 0.09), but subgroup analyses in age groups below 40 years showed a significantly increased seroprevalence of *T. gondii* IgGs in BD [OR: 1.8 (95% CI 1.10–2.89, *p* = 0.021]. Our meta-analysis indicates that *T. gondii* exposure may be a risk factor for BD in certain subpopulations.

## Introduction

Bipolar disorder (BD) is caused by a complex interplay between genetic and environmental factors. Genetic^1–3^, epidemiological^4^, imaging^5^, and molecular studies on the brain, cerebrospinal, and blood samples^6–8^ have all described an association between BD and the immune system. It has therefore been hypothesized that exposure to certain infections could be one of the environmental risk factors that contribute to developing BD. An interaction between genetic risk factors and these pathogens, or the immune response elicited by these pathogens, is thought to be involved in the dysregulation of certain neuronal circuits, underlying BD.

Neurotropic pathogens have been proposed as the main candidates for this hypothesis^9^. This includes several types of herpesviruses (herpes simplex virus (HSV)-1 and -2), Epstein-Barr virus (EBV), and cytomegalovirus (CMV), as well as the intracellular parasite *Toxoplasma gondii* (*T. gondii*). These pathogens are prevalent in humans worldwide, are capable to cross the blood–brain barrier to cause a latent infection and as such may influence CNS functions during periods of (re-)activation^10^. This neurotropic-pathogen hypothesis is supported by several lines of evidence: BD patients have been shown to have higher rates of chronic infectious diseases^11,12^; infection of the central nervous system by these neurotropic pathogens can lead to BD-like symptoms, including mania, depression, and psychosis^13,14^; exposure of animals to neurotropic pathogens, such as *T. gondii* and HSV, can induce cognitive and behavioral changes later in life^15^ and lead to neurotransmitter abnormalities (dopamine, serotonin, glutamate) that have also been associated with BD^16^.

Exposure to these infectious agents can be investigated by determining the presence of immunoglobulin M or G (IgM or IgG) class antibodies to these pathogens in blood. IgM is the first antibody to appear after infection and produced only in the first phase after exposure. IgG is produced in a delayed response to a primary infection and can generally be detected in the blood during the entire lifespan. Seroprevalence rates of IgG, therefore, represent exposure to a pathogen during life. An increase of the IgG titer level after the initial exposure can be indicative for reactivity, reinfection, or chronicity of infection. Antibody titer levels of the seropositive cases are therefore thought to correspond to the activity of the pathogen after exposure. IgG seroprevalence and titer levels have been used to study the relation between BD and these neurotropic pathogens in various previous studies^17–32^. *T. gondii* stands out as the infection with the strongest evidence. Sutterland et al. performed a meta-analysis of 11 studies that assessed the seroprevalence of *T. gondii* antibodies in BD. The authors found a significantly higher prevalence of *T. gondii* antibodies with an overall odds ratio (OR) of 1.5 (ref. ^32^), but the heterogeneity of the results was high. The mean age of participants and publication of a study in a peer-reviewed journal explained a majority of this variance. These findings are consistent with another more recent meta-analysis on eight studies showing that *T. gondii* infection is associated with BD (OR 1.26, 95% CI 1.08–1.47)^33^. Only a few studies analyzed the association between BD and IgG titers to *T. gondii*. One study found significantly higher IgG *T. gondii* titer levels in BD^27^; however, several other studies did not find any differences^24,25,34,35^. Weaker evidence is available for the neurotropic viruses. The association between BD and the seroprevalence of herpesviruses HSV-1, HSV-2, CMV is uncertain. A relation was reported for HSV-1 IgGs and cognitive functioning^20,21,30,36^, whereas CMV IgG’s were associated with hippocampus volume^22^, telomere length^19^, or certain genetic risk factors in BD^34^. However, a majority of the case–control studies did not provide evidence for a direct link between these infectious agents and BD^24,25,27,34^. Individual studies may be underpowered for reliable comparisons and the excess of nominally significant results with an almost consistent direction of higher levels of seropositivity or titer may support the infection hypothesis of BD^27,33,34^. Because of small sample sizes, correction for confounders such as age, gender, ethnicity, and other factors was not always possible in previous studies and could have contributed to inconsistent results. Another complicating factor is the variability of the prevalence rates of herpesviruses and *T. gondii* between different regions. Worldwide seroprevalences of herpesviruses and *T. gondii* vary between <5% and >95% depending on environmental and socioeconomic conditions, as well as certain habits and health-related practices^37–39^. National seroprevalence rates in the Netherlands are around 50–75% for HSV-1 (ref. ^40^), 1–25% for HSV-2 (ref. ^40^), 85–95% for EBV, 40–95% for CMV^41^, and 40% for *T. gondii*^42^.

Thus, although exposure to neurotropic pathogens has been repeatedly proposed as an environmental risk factor for BD, the supporting evidence remains inconsistent. The aim of this study was, therefore, to further examine the role of these infections. We first investigated whether the seroprevalences or titers of HSV-1, HSV-2, EBV, CMV, and *T. gondii* IgG were increased in patients with BD compared to healthy controls using plasma samples of participants of the Dutch Bipolar (DB) Cohort. Using a large number of comprehensively phenotyped BD type I patients, first-degree relatives, and healthy controls of Dutch ancestry, this study constitutes the largest number of individuals so far. To minimize the effect of possible confounders such as household, socioeconomic status, educational level, and urbanicity we included a group of matched first-degree relatives who shared a household with BD participants as “intermediate” control group and assessed whether neurotropic pathogens are associated with BD occurrence.

In order to put our results in perspective to the previous studies, we subsequently performed a systematic review and meta-analysis on all published studies that measured seroprevalences of IgG class antibodies against HSV-1, HSV-2, EBV, CMV, and *T. gondii* in well-characterized cohorts of BD patients in comparison to healthy controls. Studies were quantitatively summarized and combined in a meta-analysis where possible. Consequently, by adding new data from a large BD cohort to previous results, this study aims to provide a comprehensive and updated analysis of the evidence for an association between exposure to neurotropic pathogens and BD.

## Material and methods

### Study population

The DB Cohort study, started in 2011, is a multicenter case–control study in the Netherlands, investigating genetic and phenotypic information of patients with BD type I, first-degree relatives, and controls. Study design and recruitment procedure have been described in detail elsewhere^43^. In brief, patients with BD, their first-degree family members and healthy controls were recruited via clinicians, the Dutch BD patients’ association, pharmacies, advertisements, and through individuals who previously participated in scientific studies. Inclusion criteria for study subjects were: age ≥ 18 years, at least three Dutch-born grandparents, and a good command of the Dutch language. Subjects were excluded from analyses in case of a somatic illness that could have influenced the diagnosis of BD. Psychiatric status of BD patients was determined based on the Structured Clinical Interview for DSM-IV (SCID-I)^44^. To assess psychopathology, first-degree relatives of BD patients and healthy controls were evaluated by use of Mini-International Neuropsychiatric Interview^45^. Plasma samples were collected from a subgroup of participants of this cohort during the clinical assessments between 9 AM and 5 PM. Plasma samples were frozen and stored at −80 °C prior to determination of IgG class antibodies against HSV-1, HSV-2, EBV, CMV, and *T. gondii*. Plasma-material was available from 760 BD patients and 132 healthy controls. To minimize effects of possible confounders (household, urbanicity, socioeconomic status) on the outcome, bipolar patients (*n* = 144) were age- and gender-matched with a first-degree relative (*n* = 144) to assess whether neurotropic pathogens are associated with BD occurrence. The Medical Ethical Review Committee Utrecht approved the study (METC: 10–285). Written informed consent was obtained from all subjects after a complete description of the study was given. Demographic characteristics are displayed in Table 1 and Supplementary Table 1.

**Table 1:** Demographic characteristics, seropositivity, and IgG titer levels of HSV-1, HSV-2, CMV, EBV, and TG in bipolar disorder patients and controls.

	Patients (*n* = 760)	Controls (*N* = 132)	*p* value	*p* value adjusted^*^
Mean age (SD) in years	49.41 (12.37)	49.45 (12.80)	*p* = 0.948	
Range	18–79	18–79		
Gender M/F (% males)	335/425 (44.1)	74/85 (56.0)	*p* = 0.615	
Diagnosis	BD type I 1 748	Unipolar disorder 10		
	BD type II 12	Anxiety disorder 7		
		Other disorders^a^ 13		
		No disorder 100		
		Unknown 22		
Mean duration of illness in years	18.42 (10.9)			
Range	1–51			
**Seropositivity positive/negative cases (% positive cases)**				
HSV-1	367/393 (48.2%)	58/74 (43.9%)	*p* = 0.356	*p* = 0.374
HSV-2	71/689 (9.3%)	10/122 (7.6%)	*p* = 0.514	*p* = 0.713
CMV	325/435 (42.7%)	52/80 (39.3%)	*p* = 0.469	*p* = 0.527
EBV	480/280 (63.2%)	83/49 (62.9%)	*p* = 0.951	*p* = 0.868
TG	275/485 (36.2%)	48/84 (36.3%)	*p* = 0.968	*p* = 0.932
**Median levels (interquartile range)**				
HSV-1 IgG	34.42 (27.61)	30.46 (33.48)	*p* = 0.210	*p* = 0.257
HSV-2 IgG	25.55 (26.68)	15.56 (11.17)	*p* = 0.100	*p* = 0.116
CMV IgG	47.13 (27.13)	51.83 (24.63)	*p* = 0.443	*p* = 0.204
EBV IgG	16.31 (5.23)	17.39 (5.85)	*p* = 0.154	*p* = 0.339
TG IgG	225.74 (191.17)	229.61 (190.59)	*p* = 0.894	*p* = 0.896

^*^P value adjusted for age and gender

^a^Other disorders include ADHD, adjustment disorder, eating disorder, substance abuse

### Measurement of IgG antibodies against HSV-1, HSV-2, EBV, CMV, and *T. gondii*

IgG class antibodies against HSV-1, HSV-2, EBV, CMV, and *T. gondii* were analyzed using commercial enzyme-linked immunosorbent assay (ELISA) tests (IBL Laboratories, Hamburg, Germany) according to the manufacturer’s protocols and further described by De Witte et al.^46^. The sensitivity and specificity of the tests are above 95% for HSV-1, EBV, CMV, and *T. gondii*. For HSV-2 the sensitivity is 87.5% and specificity 94.1%. For the qualitative interpretation of the results, we used the cut-off control sample provided as standard within the ELISA kit. Samples with scores above the control cut*-*off value were scored positive and below as negative. Seropositive samples were further analyzed to determine the IgG plasma titer levels and expressed as units/ml following the manufacturer’s protocols, in which IgG levels for CMV, HSV-1, and HSV-2 are quantified by calculating (patient absorbance value × 10)/(absorbance value of the cut-off), and IgG levels for EBV and *T. gondii* are determined by using the calibration curve provided with the test and expressed as units/ml. Samples were analyzed blinded for diagnostic or clinical information.

### Statistical analysis

Statistical analysis was performed using SPSS 23.0. All variables were tested for homogeneity of variances and normality of distribution by means of the Levene and Kolgomorov–Smirnov tests, respectively. For sample characteristics, chi-square tests were used to evaluate categorical data; independent *t*-tests were performed for continuous data. The seroprevalence of IgG antibodies was analyzed as a dichotomized variable (positive/negative) with a chi-square test for BD as compared to controls. The differences in seroprevalence between BD patients and first-degree relatives were also tested using McNemar’s test. Logistic regression was performed to determine the odds for IgG positivity in BD as compared to controls and siblings while adjusting for age and gender. Plasma titers of positive cases were compared using non-parametric testing because data were non-normally distributed. Mann–Whitney *U* test was used to compare plasma titers of BD patients and controls and Wilcoxon Rank test was used to compare BD patients and their matched first-degree relatives. In order to adjust for age and gender, a post hoc ANCOVA was performed. In the case of non-normally distributed residuals, the natural log transformation (ln) was applied. Overall, ANCOVA analyses were comparable to outcomes of the non-parametric statistics. The significance level was set at *α* < 0.05 (two-tailed). Results were adjusted for multiple testing using a Bonferroni correction for ten tests.

### Literature search

This quantitative review was performed according to the Preferred Reporting Items for Systematic Reviews and Meta-Analyses (PRISMA)^47^. A systematic search was performed in Pubmed using the following search term: (bipolar OR manic* OR mania OR mood disorder) AND (virus OR virus* OR parasite OR parasit* OR toxoplasm*) (see Supplementary Material). Search cut-off date was 19 March 2019. Pre-specified inclusion criteria were: (1) human case–control studies, comparing (2) patients with a current diagnosis of BD, with the exclusion of patients with unclearly or undefined mood or affective disorders with (3) healthy controls, characterized by absence of psychiatric disorders such as affective and psychotic disorders, (4) >10 samples in each study arm; (5) measuring the seroprevalence of IgG class antibodies against HSV-1, HSV-2, EBV, CMV or *T. gondii*; (6) original research, published in a peer-reviewed journal; (7) written in English. In the case of follow-up data or overlapping samples, we included outcomes of the largest sample size. A total of 1954 studies were found. Three authors (G.J.L.J.S., H.C.v.M., and L.D.d.W.) independently performed the database searches, reviewed title and abstracts, and selected the articles that appeared to meet the inclusion criteria. Two independent authors reviewed each of the selected manuscripts in full (G.J.L.J.S. and L.D.d.W.) (see Supplementary Fig. 1 and Supplementary Table 4). When seroprevalence rates of IgG class antibodies against HSV-1, HSV-2, EBV, CMV, or *T. gondii* were not given in the original article, we contacted the corresponding author asking if we could obtain raw data. After 2 weeks, a reminder was sent to authors who did not respond. Reference lists of retrieved articles and two recently published meta-analyses were screened for additional relevant articles (*n* = 10) and the present study was included in the meta-analysis. See Supplementary Fig. 1.

### Meta-analysis

All analyses were carried out using the Comprehensive Meta-Analysis^48^ software developed by Biostat. Five or more studies were minimally required for meta-analysis. Unadjusted odds ratio’s (OR) was used to quantify effect sizes (ES) for the differences between bipolar patients vs. a control group. Given the heterogeneity among studies, we used a random effects model for all comparisons^49^. The *Q*-statistic test, displaying a chi-square distribution with k-1 degrees of freedom, was performed to evaluate the existence of heterogeneity. Higher *Q*-values than degrees of freedom indicates that the variability among studies is higher than would be expected due to randomness and further examination of subgroups is warranted. *I*-squared (*I*^2^) was calculated to estimate the amount of heterogeneity. *I*^2^ reflects which proportion of the observed variance reflects differences in true effect size rather than sampling error (range 0–100%), with an *I*^2^ of 25% indicating low, 50% moderate, and more than 75% high heterogeneity^50^. The potential for publication bias was assessed by visual examination of Funnel plots and by the Egger’s test (which was considered significant if the one-sided *p* value was ≤0.10)^51^. As ethnicity, methodology, age, and gender were expected to vary between studies, we investigated which moderators were associated with the reported OR’s using random effects meta-regression analyses. In the case of significant outcomes (*p* < 0.05), subgroup analyses were performed. Random effects meta-regression analyses were conducted. We conducted “one study removed” and “outlier removed” sensitivity analyses^52^. A-one-study removed sensitivity analysis was performed by iteratively removing one study at a time to confirm that our findings were not driven by a single study^48^. To reduce the amount of heterogeneity, outlying studies were removed and the meta-analysis was performed again^53^. Potential outlier studies were defined as standardized residual *z*-scores of effect sizes exceeding ±1.96 (*p* ≤ 0.05 two-tailed).

### Quality check

Several criteria were assessed to evaluate the quality of the studies that were included in our meta-analysis. Definition of the study population was qualified as high (+) when both the patient and control groups were well described; a structured clinical interview was used to confirm the diagnosis in patients and exclude psychopathology in controls, and the study population reflects the source population. The methodological standards were defined as high when laboratory tests to measure IgGs and cut-offs were well described. Blinded outcome measurements were qualified as high when raters were blind to diagnostic or clinical information. Correction for confounding factors was scored high when studies corrected for multiple relevant confounding factors and intermediate (+/−) when correction for only age and gender was applied. Selection bias refers to systematic differences between the baseline characteristics of the groups that were compared. This was rated high if groups were comparable and low if baseline characteristics were different. Selective reporting is qualified low when important details regarding the study population or outcome “seroprevalence” could not be retrieved from the study. We identified the following potential confounders (age, gender, ethnicity, psychotropic medication, disease state, socioeconomic, household, and lifestyle factors). We checked the description of these variables and whether they were restricted for by matching or statistical analyses in the included studies (see Supplementary Table 3).

## Results

### DB Cohort

Characteristics of included BD patients and controls are summarized in Table 1. Seroprevalence rates for HSV-1 [adjusted OR 0.84, CI 95% 0.57–1.23], HSV-2 [adjusted OR 0.88, 95% CI 0.44–1.76], CMV [adjusted OR 0.88, 95% CI 0.60–1.30], EBV [adjusted OR 0.97, 95% CI 0.66–1.42], and *T. gondii* [adjusted OR 1.02, 95% CI 0.67–1.54] showed no significant differences between BD patients and controls while adjusting for age and gender. IgG titer levels of HSV-1, HSV-2, CMV, EBV, and *T. gondii* did not significantly differ between BD patients and controls (see Table 1). However, the sample size of the control group is considerably smaller (*n* = 132) than the BD group (*n* = 760). To increase our control group to *n* = 400, we included 268 healthy controls of Dutch ancestry from the genetic outcome risk of psychosis cohort. The seroprevalence and titers of this cohort were previously tested using the same method and described before^46^. Increased seroprevalences or titers were not found when the BD group was compared to the pooled control group with adjustments for age and gender (data not shown). No significant differences were detected comparing the presence of any of the five neurotropic pathogens in BD patients vs. controls [adjusted OR 1.322, 95% CI 0.706–2.475].

As shown in Table 2 no significant differences between BD patients and matched first-degree relatives were identified with regard to IgG class antibodies titers against HSV-1, HSV-2, EBV, CMV, and *T. gondii*. We found that the seroprevalence of HSV-2 is significantly higher in BD patients (8.3% vs. 4.2%, *p* < 0.001) and HSV-2 IgG titers were higher (median 33.10 units/ml (IQR 32.15) vs. 20.47 units/ml (IQR 11.96), *p* = 0.131, *p* corr = 0.043) as compared to first-degree relatives. However, the significant difference in IgG titer level did not survive Bonferroni correction. Exclusion of control subjects or first-degree relatives diagnosed with a unipolar depression does not alter the results (data not shown).

**Table 2:** Demographic characteristics, seropositivity, and IgG titer levels of HSV-1, HSV-2, CMV, EBV, and TG in bipolar disorder patients and matched first-degree relatives.

	Patients (*N* = 144)	Matched first-degree family members (*n* = 144)	*p* value^a^	*p* value pairwise^b^	*p* value adjusted^c^
Mean age (SD) in years	48.05 (12.75)	54.14 (15.35)	*p* = 0.000		
Range	23–79	18–88			
Gender M/F (% males)	49/95 (34.0)	45/99 (31.25)	*p* = 0.615		
Diagnosis	BD type I 143	Anxiety disorder 9			
	BD type II 1	Depression 30			
		Psychotic disorder 1			
		Other disorders* 13			
		No disorder 92			
Mean duration of illness in years	18.0 (11.6)				
Range	1–51				
**Seropositivity positive/negative cases (% positive cases)**					
HSV-1	62/82 (43.0%)	72/72 (50.0%)	*p* = 0.237	*p* = 0.468	*p* = 0.669
HSV-2	12/132 (8.3%)	6/138 (4.2%)	*p* = 0.144	*p* < 0.001	*p* = 0.152
CMV	55/89 (38.2%)	68/76 (47.2%)	*p* = 0.121	*p* = 0.110	*p* = 0.319
EBV	79/65 (54.9%)	77/67 (53.5%)	*p* = 0.813	*p* = 0.356	*p* = 0.678
TG	44/100 (30.5%)	64/80 (44.4%)	*p* = 0.015	*p* = 0.006	*p* = 0.266
**Median levels (interquartile range)**					
HSV-1 IgG	30.75 (27.09)	33.72 (24.15)	*p* = 0.386	*p* = 0.184	*p* = 0.091
HSV-2 IgG	33.10 (32.15)	20.47 (11.96)	*p* = 0.131	NA	*p* = 0.043
CMV IgG	45.53 (26.56)	51.05 (22.96)	*p* = 0.314	*p* = 0.228	*p* = 0.636
EBV IgG	15.78 (5.41)	16.13 (5.28)	*p* = 0.337	*p* = 0.771	*p* = 0.803
TG IgG	212.97 (182.76)	190.06 (204.03)	*p* = 0.845	*p* = 0.426	*p* = 0.726

*NA* not applicable ^a^Comparison using chi-squared test or Mann–Whitney *U* test ^b^Paired comparison using McNemar’s test or Wilcoxon Rank test ^c^*P* value adjusted for age and gender

### Systematic search

The flowchart in Supplementary Fig. 1. depicts the results of our systematic search. Fourteen studies investigating IgG class antibodies against HSV-1, HSV-2, CMV, *T. gondii*, and/or EBV were identified, including a total of 2364 BD patients and 5101 healthy controls. The age range for the included studies was between 30 and 57 years. Details on the methodological design, number of participants, and outcome measures for the individual studies are described in Table 3. A quality assessment of the studies is provided in Supplementary Table 2.

**Table 3:** Summary of included studies.

Pathogen	Author	Year	Country (ethnicity)	Patients	Controls	Technique	Age patients (mean (SD), years)	Age controls (mean (SD), years)	Gender patients (male, *N* (%)	Gender controls (male, *N* (%)	Seropositive patients positive, *N* (%)	Seropositive controls positive, *N* (%)	Significant
CMV	Avramopoulos	2015	US (Jewish)	489	362	ELISA	43.3 (18.0)	57.9 (12.2)	235 (48.1)	148 (40.9)	157 (32.1)	128 (35.3)	N
	Dickerson	2004	US	117	100	Solid phase immunoassay	41.4 (12.2)	36.0 (13.3)	35 (30.0)	25 (25.0)	42 (35.9)	17 (17)	Y
	Gerber	2012	Germany	30	20	Solid phase immunoassay	42.6 (NA)	39.8 (NA)	12 (40.0)	7 (35.0)	9 (30.0)	5 (25.0)	N
	Hamdani	2017	France	138	180	Solid phase immunoassay	44.3 (13.3)	40.1 (13.8)	(65 (47.1)	98 (54.4)	79 (57.2)	119 (66.1)	N
	Prossin	2015	US	139	99	Solid phase immunoassay	39.0 (13.0)	32 (14.0)	52 (37.0)	49 (49.0)	77 (55.3)	40 (40.4)	Y
	Rizzo	2013	Brazil	22	17	Chemiluminescent enzyme immunometric assays	44.62 (9.30)	39.47 (12.89)	0 (0.0)	0 (0.0)	20 (90.9)	15 (88.2)	N
	Snijders	Present study	The Netherlands (Dutch)	760	132	ELISA	49.41 (12.37)	49.45 (12.80)	335 (44.1)	74 (56.0)	325 (42.7)	52 (39.9)	N
	Tanaka	2017	US	32	32	ELISA	38.72 (7.46)	38.16 (7.08)	14 (43.8)	14 (43.8)	22 (68.8)	22 (68.8)	N
	Tedla	2011	Ethiopia	199	80	ELISA	31.6 (NA)	30.4 (NA)	105 (52.8)	53 (66.2)	198 (99.5)	80 (100.0)	N
HSV-1	Avramopoulos	2015	US (Jewish)	489	362	ELISA	43.3 (18.0)	57.9 (12.2)	235 (48.1)	148 (40.9)	178 (36.4)	144 (39.8)	N
	Dickerson	2004	US	117	100	Solid phase immunoassay	41.4 (12.2)	36.0 (13.3)	35 (30.0)	25 (25.0)	49 (41.9)	46 (46.0)	N
	Gerber	2012	Germany	30	20	Solid phase immunoassay	42.6 (NA)	39.8 (NA)	12 (40.0)	7 (35.0)	16 (53.3)	10 (50.0)	N
	Hamdani	2017	France	138	180	Solid phase immunoassay	44.3 (13.3)	40.1 (13.8)	(65 (47.1)	98 (54.4)	87 (63.0)	130 (72.2)	N
	Snijders	Present study	The Netherlands (Dutch)	760	132	ELISA	49.41 (12.37)	49.45 (12.80)	335 (44.1)	74 (56.0)	367 (48.2)	58 (43.9)	N
	Tanaka	2017	US	32	32	ELISA	38.72 (7.46)	38.16 (7.08)	14 (43.8)	14 (43.8)	22 (68.8)	18 (56.3)	N
	Tedla	2011	Ethiopia	199	80	ELISA	31.6 (NA)	30.4 (NA)	105 (52.8)	53 (66.2)	191 (96.0)	80 (100.0)	N
HSV-2	Dickerson	2004	US	117	100	Solid phase immunoassay	41.4 (12.2)	36.0 (13.3)	35 (30.0)	25 (25.0)	35 (29.9)	20 (20.0)	N
	Gerber	2012	Germany	30	20	Solid phase immunoassay	42.6 (NA)	39.8 (NA)	12 (40.0)	7 (35.0)	4 (13.3)	1 (5.0)	N
	Hamdani	2017	France	138	180	Solid phase immunoassay	44.3 (13.3)	40.1 (13.8)	(65 (47.1)	98 (54.4)	33 (23.9)	51 (28.3)	N
	Snijders	Present study	The Netherlands (Dutch)	760	132	ELISA	49.41 (12.37)	49.45 (12.80)	335 (44.1)	74 (56.0)	71 (9.3)	10 (7.6)	N
	Tedla	2011	Ethiopia	199	80	ELISA	31.6 (NA)	30.4 (NA)	105 (52.8)	53 (66.2)	30 (15.1)	8 (10.0)	N
*T. gondii*	Abdollahian	2017	Iran	70	350	ELISA	NA	38.0 (13.2)	NA	170 (48.5)	33 (47.1)	120 (34.3)	Y
	Avramopoulos	2015	US (Jewish)	489	362	ELISA	43.3 (18.0)	57.9 (12.2)	235 (48.1)	148 (40.9)	57 (11.7)	72 (19.9)	Y
	Chen	2019	China	115	681	ECLIA	37.6 (12.2)	37.7 (0.53)	56 (48.7)	381 (55.9)	22 (19.1)	64 (9.4)	Y
	Gerber	2012	Germany	30	20	Solid phase immunoassay	42.6 (NA)	39.8 (NA)	12 (40.0)	7 (35.0)	4 (13.3)	1 (5.0)	N
	Hamdani	2017	France	138	180	Solid phase immunoassay	44.3 (13.3)	40.1 (13.8)	65 (47.1)	98 (54.4)	103 (74.6)	105 (58.3)	Y
	Hinze-Selch	2010	Germany	87	214	Indirect immunofluorescence	46.3 (14.0)	38.9 (13.3)	NA	NA	41 (47.1)	86 (40.1)	N
	Khadamavetan	2013	Iran	117	200	ELISA	33.93 (11.9)	33.88 (11.45)	59 (50.4)	96 (48.0)	37 (31.6)	53 (26.5)	N
	Tanaka	2017	US	32	32	ELISA	38.2 (7.1)	38.7 (7.5)	14 (43.8)	14 (43.8)	7 (21.8)	6 (18.8)	N
	Tedla	2011	Ethiopia	171	71	ELISA	31.6 (NA)	30.4 (NA)	105 (52.8)	53 (66.2)	163 (95.3)	62 (87.3)	Y
	Snijders	Present study	The Netherlands (Dutch)	760	132	ELISA	49.41 (12.37)	49.45 (12.80)	335 (44.1)	74 (56.0)	275 (36.2)	48 (36.3)	N
	Xiao	2015	China	49	2634	ELISA	NA	NA	NA	1319 (50.0)	5 (10.2)	329 (12.5)	N
EBV	Dickerson	2004	US	117	100	Solid phase immunoassay	41.4 (12.2)	36.0 (13.3)	35 (30.0)	25 (25.0)	89 (76.1)	87 (87.0)	N
	Snijders	Present study	The Netherlands (Dutch)	760	132	ELISA	49.41 (12.37)	49.45 (12.80)	335 (44.1)	74 (56.0)	480 (63.2)	83 (62.9)	N

*CMV* cytomegalovirus, *EBV* Epstein-barr virus, *ECLIA* electrochemiluminescence immunoassay analyzer, *ELISA* enzyme-linked immunosorbent assay, *HSV-1* herpes simplex virus-1, *HSV-2* herpes simplex virus-2, *N* no, *Y* yes, *T. gondii* *Toxoplasma gondii*, *SD* standard deviation, *US* United States

### Meta-analyses herpesviruses

**Fig. 1:**
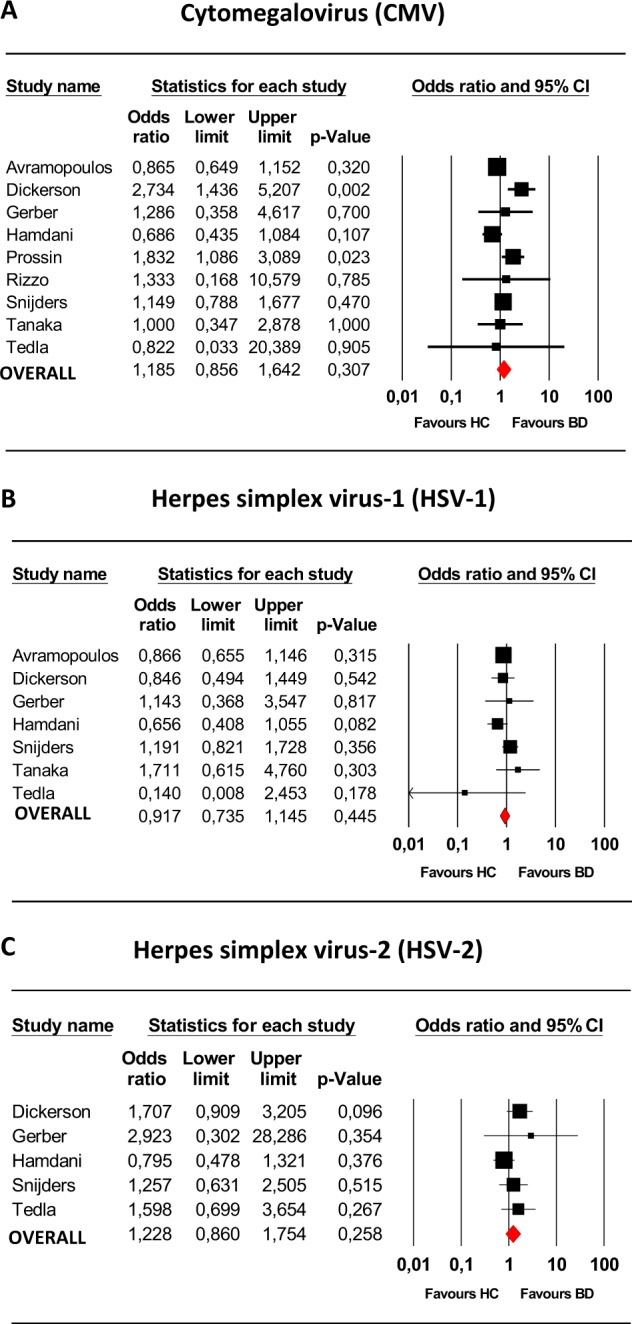
Bipolar disorder and prevalence of IgG antibodies against Cytomegalovirus, Herpes simplex virus-1, and Herpes simples-virus-2. Meta-analysis on **a** cytomegalovirus (CMV) (*n* = 9), **b** herpes simplex virus-1 (HSV-1) (*n* = 7), **c** herpes simplex virus-2 (HSV-2) (*n* = 5) in bipolar disorder. BD bipolar disorder, HC healthy controls.

Our systematic search resulted in the inclusion of nine studies for CMV, seven for HSV-1, five for HSV-2, and two for EBV (Table 3). A meta-analysis was performed for CMV, HSV-1, and HSV-2. Figure 1a–c shows that the seroprevalence of IgG was not significantly increased in BD for these three viruses (CMV: OR = 1.19, CI 95% 0.85–1.64, *p* = 0.30; HSV-1: OR 0.92, 95% CI 0.73–1.14, *p* = 0.44; HSV-2: OR 1.22, CI 95% 0.86–1.75, *p* = 0.25). Inspection of the funnel plots and the Egger’s test did not show indications for publication bias. The heterogeneity for the studies on HSV-1 and HSV-2 was low (*I*^2^ = 18% respectively *I*^2^ = 17%), but for CMV heterogeneity was moderate-high: *I*^2^ = 56%. The *Q*-value (*p* < 0.02) indicated significant variability between studies for CMV. The number of studies on EBV was too low to perform a meta-analysis. The individual OR of the study of Dickerson et al.^20^ just reached significance (*p* = 0.04), but the present study was not significant (*p* = 0.95). We analyzed several moderators that could potentially have an influence on CMV, EBV, HSV-1, and HSV-2 seroprevalences, including gender, age, the seroprevalence of the control group, ethnicity (Caucasian or not) or measurement technique. None of these factors were associated with the OR or significantly changed heterogeneity for any of the herpesviruses. No potential outlier studies were detected for herpesviruses. Sensitivity analyses with the removal of the DB Cohort or the other studies showed that removal of the study of Hamdani et al.^26^ revealed a significant association between HSV-2 and BD (OR 1.54, 95% CI 1.03–2.30, *p* = 0.033). Removal of other studies did not show significant results.

### Meta-analysis *T. gondii*

**Fig. 2:**
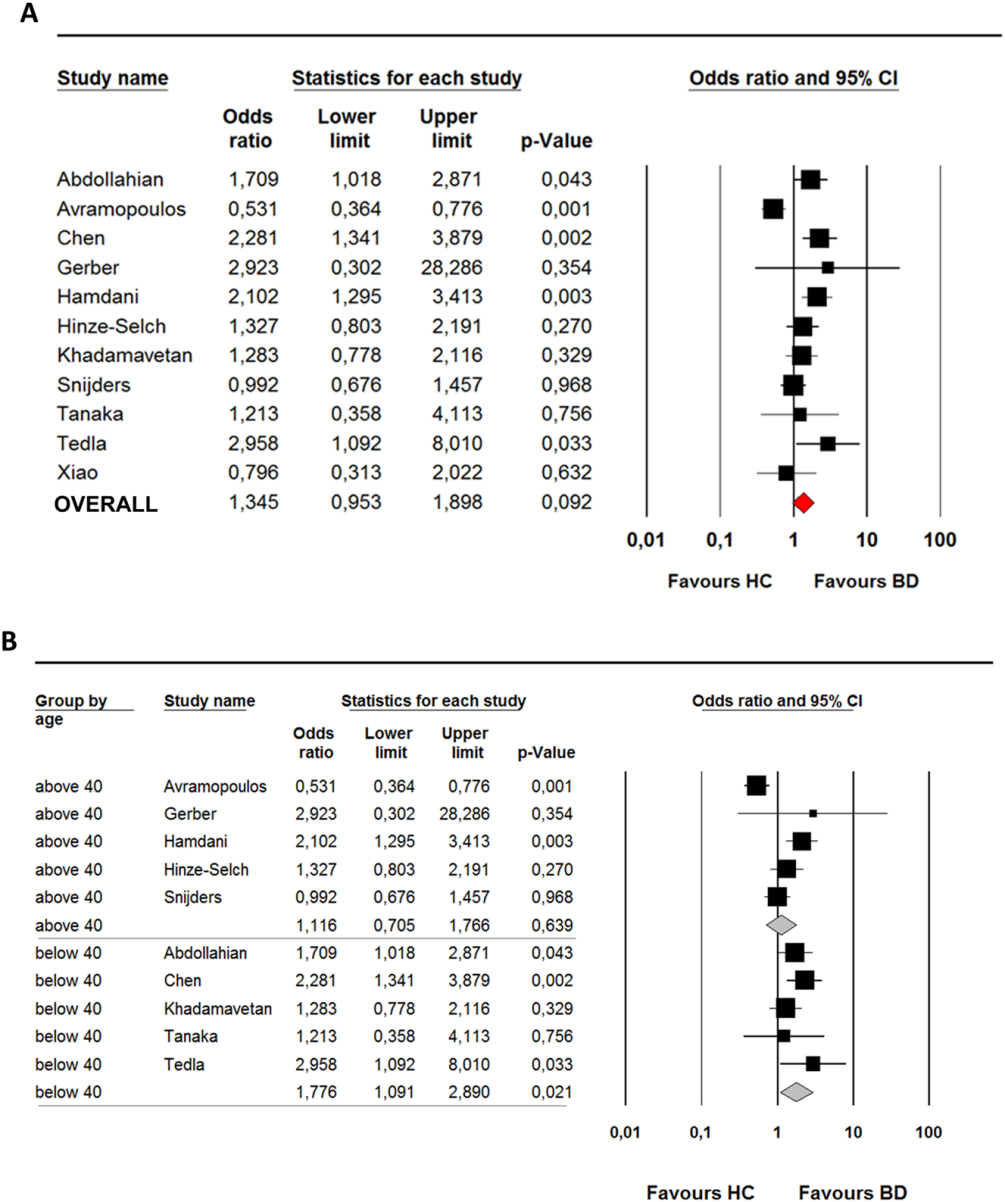
Bipolar disorder and prevalence of IgG antibodies against *T. gondii*. **a** Meta-analysis on Toxoplasma gondii (*T. Gondii*) (*n* = 11) in bipolar disorder. **b** Subgroup analysis (age > 40 years and age < 40 years) on Toxoplasma gondii (*T. gondii*) in bipolar disorder (*n* = 10). Two age categories were based on the mean age (in years) of the included studies in the meta-analysis (<40 years and >40 years).

Eleven studies met the inclusion criteria of our systematic search for *T. gondii*. Weight of the included studies was largely similar (ranging from 5% to 12%), except for the study of Gerber et al.^21^. The quality assessment of the studies is provided in Supplementary Table 2. The majority of the studies, but not all, matched or corrected for age and gender and additional confounders relevant to *T. gondii* infection, including ethnicity, socioeconomic, lifestyle and household factors^54^. Only the studies of Hamdani^26^, Tedla^24^, and the present study in DB Cohort used structured interviews to confirm a psychiatric diagnosis in the patient group and exclude psychopathology in the control group. Serology assessments were in general comparable between studies and often performed at the same laboratory^18,20,21,25,34^. The overall OR of the meta-analysis was not significant (OR 1.4 95% CI 0.95–1.90, *p* = 0.09) and the heterogeneity was high (*I*^2^ = 72%). The *Q*-value (*p* < 0.001) indicated significant variability between studies (see Fig. 2a). There was no evidence of publication bias with Egger’s test (*p* = 0.12), confirmed by examination of the funnel plot. Removal of the DB Cohort did not change the results (overall OR 1.41, 95% CI 0.95–2.10, *p* = 0.09). Removal of the “negative” study of Avramopoulos revealed a significant positive association between *T. gondii* and BD (OR 1.5, 95% CI 1.18–1.92, *p* = 0.001). Two positive^26,29^ and one negative^34^ studies were identified as potential outliers. Removal of these three potential outliers resulted in a significant positive association between *T. gondii* and BD (OR 1.28, 95% CI 1.03–1.92, *p* = 0.03).

### Potential moderators *T. gondii*

To assess which moderators influenced the outcome of the meta-analysis, we first investigated which moderators were associated with the reported ORs in the included studies. No significant relation of ethnicity, male gender, seroprevalence rates of the control population, and measurement technique with the value of the OR was found. For mean age (as reported by 10 of the 11 studies) a negative association was observed (intercept = 2, 82, slope = −0.06, *p* = 0.003). A subgroup analysis was performed post hoc. At first, the study samples were divided into two age categories based on the mean age (in years) of the studies included in the meta-analysis (<40 years and >40 years).

Significant higher seroprevalence of *T. gondii* was found for BD in age group <40 years with an overall OR of 1.8 (95% CI 1.10–2.89, *Q* (4) = 3.8, *p* = 0.021, *I*^2^ = 0%). Heterogeneity was absent in this subgroup, although the forest plot showed considerable variation in ORs. No significant differences were found in the group aged >40 years and overall OR was 1.1 (95% CI 0.70–1.76, *Q* (4) = 21.8, *p* = 0.64, *I*^2^ = 82%) (see Fig. 2b). The differences in OR between the two age groups was not significant (*p* = 0.174).

## Discussion

In this study, we investigated whether infection with neurotropic pathogens could be one of the environmental factors that in interaction with genetic background contributes to BD. The seroprevalence, which reflects prior exposure to a certain pathogen, of none of these IgGs was increased in BD patients as compared to healthy controls or siblings in the DB Cohort. The titer levels of IgGs to these pathogens were also not increased in patients compared to controls or siblings, although we found higher HSV-2 seroprevalence rates and titers in patients compared to siblings only. To draw a more general conclusion on the results of this and previous studies we subsequently performed a systematic review and meta-analysis for the seroprevalences of these pathogens in BD.

The hypothesis of neurotropic pathogens as causing or contributing factor in BD was based on studies that have described immune alterations in BD^1–8^. Low-grade inflammation has been documented in BD. Increased levels of pro-inflammatory cytokines and acute phase proteins have been shown during acute mood episodes and in later stages of the disease^55–60^. Furthermore, studies have shown antidepressant effects of anti-inflammatory medication in the treatment of a bipolar depression^61^. Neurotropic pathogens may be one factor that plays a role in this inflammatory process through activation of the adaptive and innate immune systems. This phenomenon has been described extensively for schizophrenia^62^, in particular in specific subgroups of patients (cognitive impairers, smokers)^23,63,64^.

Our results on the seroprevalences of herpesviruses in the DB Cohort are in line with the findings from the meta-analysis, showing no association between BD and IgG prevalence to any of these viruses. For HSV-1 and HSV-2 none of the previous studies showed a significant association with BD. The heterogeneity of results on these viruses is low, with the exception of CMV. Two previous studies with a moderate sample size found an increased seroprevalence of CMV^18,20^. Our current analysis and meta-analysis did not confirm this association. In the studies of Prossin and Dickerson considerable differences between groups were reported for age, ethnicity, and gender. It is not clear from the methodology whether adjustments for these potential confounders were made. Sensitivity analyses showed that the removal of the study of Hamdani et al.^26^ revealed a significant positive association between HSV-2 and BD. We compared study design, age, gender, methodology, and ethnicity between the study of Hamdani and the other studies. We did not observe obvious reasons for these contrasting results.

Our results of no increased seroprevalence of *T. gondii* in BD were in line with the results of our meta-analysis. We did, however, detect a high heterogeneity between studies. We found that age was significantly associated with the ORs in the different studies and explained part of the heterogeneity. The negative association between OR and age suggest that only in younger cohorts a significant effect could be detected and that this effect is lost when the seroprevalence of *T. gondii* increases in an aging population. We could confirm this hypothesis by a post hoc subgroup analysis in age groups <40 years and >40 years, but could not validate this hypothesis by analyzing a younger subgroup of <40 years (*n* = 411) in the DB Cohort (data not shown). It would, however, be interesting to examine a high risk or first-episode BD cohort for *T. gondii* IgGs. Three studies were “potential” outliers^26,29,34^ and removal of these studies resulted in a significant positive association between *T. gondii* and BD. We compared these three potential outlier studies in order to explain differences in OR. The seroprevalence of *T. gondii* was considerably higher in the study of Hamdani (74.6%)^26^, compared to the study of Avramopoulos (11.7%)^34^. The difference between the two groups in seroprevalence rates may be explained by differences in the two populations in risk behavior for acquiring *T. gondii* such as ingestion of raw meat and unpasteurized milk products, as well as contacts with cats. Most *T. gondii* studies did not restrict for these factors. Furthermore, Hamdani et al.^26^ and Chen et al.^29^ included control participants that were on average 18 years younger than the controls in the study of Avramopoulos. This finding underscores the hypothesis that only in younger cohorts a significant effect may be detected, which is in line with findings in our meta-analysis. Previous literature suggests that *T. gondii* exposure is only a significant risk factor in regions with a high seroprevalence^32^. This could be caused by regional differences in age of exposure, which is lower in regions with higher seroprevalences, as well as the type of *T. gondii* strain. However, in our meta-analysis, we could not replicate the significant relation between the moderator seroprevalence rates of the control population and the OR.

Sutterland et al.^32^ and de Barros et al.^33^ previously performed a meta-analysis on *T. gondii* seroprevalences in BD and found a significant odds ratio of 1.52 (95% CI 1.06–2.18, *p* = 0.002) and 1.26 (95% CI 1.08–1.47), respectively^32,33^. In these studies, the heterogeneity was also high (*I*^2^ = 67% and *I*^2^ = 55%, respectively). Sutterland et al. (2015) showed that the high heterogeneity was mostly explained by (un)published data and age adjustment. Similar to our meta-analysis, no signs of publication bias were observed by Sutterland and colleagues. Only a few studies overlapped between the three meta-analyses. We included several studies with a large sample size that were published after the meta-analysis of Sutterland et al. and de Barros et al. (2017)^25,26,28,29^ and only included published papers, studies with sample sizes >10 and case–control studies. Sutterland et al.^32^ included unpublished studies and studies analyzing neonatal blood spots, which examines *T. gondii* antibody status of the mother of patients with BD. De Barros et al.^33^ 2017 included studies with overlapping samples. Altogether, these differences may explain the low overlap of included studies and differences in results between the three meta-analyses on *T. gondii*.

Additionally, we did not find an association between IgG titer levels to neurotropic pathogens and BD. We found higher seroprevalence rates and HSV-2 titers in patients compared to first-degree relatives, but not to controls. Six other studies have assessed HSV-2 and found no significant differences in seroprevalence or titers between patients and controls only^17,18,20,21,24,26^ (see Supplementary Table 5). A possible explanation is that bipolar persons are more likely to engage in risky sexual behavior that places them at risk of sexually transmitted infections such as HSV-2 (ref. ^65^). The inconsistent results could be explained by small sample sizes, especially the measurement of IgG titer is based on subjects that are positive for antibodies, which is low for HSV-2. Previous studies measured titer levels in the total study population, while we assessed the titer level only in seropositive cases. Therefore, a direct comparison of titer levels between studies should be interpreted with some caution. Our results are in contrast to two recent other positive studies on CMV titers with smaller sample sizes^18,19^. Rizzo et al. showed increased IgG titers in euthymic patients with BD, while Prossin et al. mentioned that CMV IgG was higher in BD type I patients with elevated moods compared to euthymic BD patients. CMV titers rise steeply between the age of 41 and 50 years in the general population^66^. In the present study, the included BD and HC participants were 5–10 years older than participants included in the two previous studies. We cannot exclude that CMV titer level differences between BD and HC subjects have diminished over time. Our results are in line with four other large studies on *T. gondii*^20,24,25,34^. Only one smaller study, performed in France, showed a significant difference in *T. gondii* IgG titer levels between groups^27^. The significant higher titer levels in the BD group might be attributed to the higher mean age in this group compared to controls (44 and 38 years, respectively). Previous studies showed that T. *gondii* infection increased significantly after 40 years of age in the French population^67^. Due to the differences in titers with age, future studies should analyze titer levels stratified for age. Another point of importance is the assumption that higher titer levels are indicative for reactivity, reinfection or chronicity of infection. Since our data are cross-sectional, we cannot distinguish between these different processes.

Strong aspects of our antibody analyses in the DB Cohort are the sample size of the patients, which is the largest to date and that we controlled for age, gender, and ethnicity. Furthermore, the inclusion of a group of first-degree relatives as “intermediate” control group also minimizes the effects of possible confounders on seropositivity as many potential confounders such as ethnicity, household, educational level, and urbanicity are shared. Limitations of our study are the small sample size of the healthy controls that were included from the DB Cohort. To overcome this problem we included controls from a previous study which used a similar methodology except for the time of sample collection and testing. Like most previous studies, we did not analyze data on several potential confounders, such as the size of household, urbanicity, contact with felines, and raw meat consumption. Moreover, we analyzed previous exposure to pathogens by analyzing IgG in blood years after disease onset. Since it is hypothesized that these pathogens play a role in the development of BD, it would be interesting to repeat this study in first-episode BD patients and include the measurement of IgM type antibodies.

We limited the study to five pathogens; therefore, this study cannot exclude a role for other neurotropic pathogens. Another strong aspect of our study is the addition of a systematic review and meta-analysis, which provides an overview of the current evidence on the association between exposure to neurotropic pathogens and BD. In contrast with previous meta-analyses, we carefully selected case–control studies with at least moderate sample sizes and excluded cohort studies which assessed the seroprevalence of mothers. However, this part of our study also has several limitations. First of all, we were unable to retrieve all relevant data, as not all authors responded to our request for additional information. Second, despite careful analysis of possible moderators, significant heterogeneity remained for the meta-analysis on CMV and *T. gondii*, which suggest that other moderators play a role. This could include differences in laboratory methodologies, such as assays used or cut-off values applied, but also other confounders related to exposure to these pathogens. Low educational level, having children, crowding, low household income and factors related to lifestyle (raw meat consumption, contact with felines, and promiscuity) are risk factors for an increased seroprevalence of neurotropic pathogens^68–70^. Several clinical variables should also be taken into account, such as disease state and severity, use of (psychotropic) medication, and duration of illness. Unfortunately, we could not analyze these factors in the meta-analysis due to inconsequent reporting and limited data. Finally, the data incorporated in the present meta-analysis might be incomplete, since we decided not to include unpublished data as these studies are not peer-reviewed and their internal validity may be difficult to assess (due to poor reporting).

In conclusion, the data presented in this study did not provide robust evidence for an association between exposure to herpesviruses and BD. Findings on *T. gondii* IgGs suggest that there is no association between exposure to this pathogen and BD, however additional studies are needed in early-onset BD patients and strain-specific analysis to determine whether T*. gondii* exposure is an environmental risk factor for BD or not. Further clarifying this potential association is of importance, since the link between *T. gondii* and BD is not only interesting for understanding the pathogenesis of BD but may also provide a target for treatment^71^.

## Supplementary information

Supplementary material
