# Supplementary material for: The association between antibodies to neurotropic pathogens and bipolar disorder: A study in the Dutch Bipolar (DB) Cohort and meta-analysis
Source: Transl Psychiatry. 2019 Nov 20;9:311. doi: 10.1038/s41398-019-0636-x (PMC6868237; doi:10.1038/s41398-019-0636-x)
Supplement: Supplementary file 1 — Supplementary material [file 41398_2019_636_MOESM1_ESM.docx]

**Supplementary Material** - **Standardized Search Algorithms**

**#PUBMED (up to March 19^th^ 2019)**

((bipolar OR manic* OR mania OR mood disorder) AND (virus OR virus* OR parasite OR parasit* OR toxoplasm*).)

**Supplementary Figure 1 Flowchart**

**
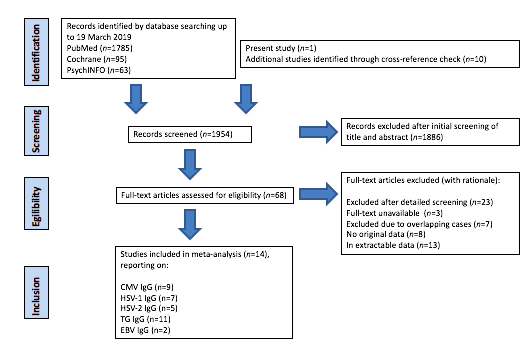
**

**Abberations** CMV= Cytomegalovirus , EBV = Epstein-barr virus, HSV-1= Herpes-simplex virus 1, HSV 2=Herpes simplex virus-2 TG= Toxoplasma *gondii* **.** IgG = Immunoglobuline G

**Supplementary Table 1 Demographic characteristics bipolar patients (*n*=760)**

| Psychotropic medication in BD patients* |
| --- |
| Lithium   - Current use (n=519) - No current use (n=241) |
| Carbamazepine   - Current use (n=77) - No current use (n=683) |
| Valproate   - Current use (n=179) - No current use (n=581) |
| Disease state in BD patients* |
| Rapid cycling   - Rapid cycling (n=97) - No rapid cycling (n=612) |
| Current mood episode   - Depressed (n=62) - Hypomanic (n=47) - Manic (n=23) - Mixed (n=4) - Unknown (n=624) |
| Severity current episode   - Mild (n=36) - Moderate (n=57) - Severe without psychosis (n=24) - Severe with psychosis (n=9) - Partial remission (n=143) - Remission (n=456) |
| Recovery between episodes   - Full recovery (n=358) - Partial recovery (n=231) |
| Last mood episode   - Depressed (n=360) - Hypomanic (n=81) - Manic (n=226) - Mixed (n=19) - Unknown (n=74) |
| Age of onset in years   - ≤29 (n=383) - >29 (n=348) |
| Disease duration in years   - 0-10 (n=182) - 10-20 (n=257) - 20-30 (n=180) - >30 (n=113) |
| Number of episodes   - <5 (n=280) - 5-10 (n=199) - 10-20 (n=89) - >20 (n=167) |
| Global assessment of functioning   - 100-91 (n=3) - 90-81 (n=34) - 80-71 (n=180) - 70-61 (n=254) - 60-51 (n=154) - <50 (n=111) |
| ASRM   - ≤6 (n=655) - >6 mania (n=90) |
| IDS   - 0-13 no depression (n=367) - 14-25 mild (n=225) - 26-38 moderate (n=111) - 39-48 severe (n=29) - 49-84 very severe (n=11) |

**Abbrevations.** ASRM: Altman Self-Rating Mania Scale. IDS: Inventory of Depressive Symptomatology.

All cut-off points are based on medians. *Clinical data was not available for all included BD subjects for the IgG analyses.

**Supplementary Table 2 Quality assessment**

| **Study** | **Year** | **Definition study participants ^a^** | **Methodology standardization^b^** | **Blinded outcome assessment^c^** | **Selection bias^d^** | **Confounding factors^e^** | **Reporting bias^f^** |
| --- | --- | --- | --- | --- | --- | --- | --- |
| **Avramopoulos ^1^** | 2015 | - | + | + | - | +/- | + |
| **Chen ^63^** | 2019 | +/- | +/- | - | +/- | +/- | + |
| **Dickerson ^2^** | 2004 | + | + | + | +/- | - | - |
| **Gerber ^3^** | 2012 | +/- | + | - | + | - | + |
| **Hamdani ^4^** | 2017 | + | + | + | +/- | + | - |
| **Prossin ^5^** | 2015 | + | + | - | + | + | + |
| **Rizzo ^6^** | 2013 | + | + | - | + | +/- |  |
| **Snijders** | 2019 | + | + | + | + | +/- | + |
| **Tanaka ^7^** | 2017 | +/- | - | - | +/- | +/- | - |
| **Tedla ^8^** | 2011 | + | + | - | + | +/- | + |
| **Abdollahian ^9^** | 2017 | +/- | + | + | + | - | +/- |
| **Hinze-Selch ^10^** | 2010 | +/- | + | - | - | - | + |
| **Khadamavetan ^11^** | 2013 | +/- | - | - | + | +/- | + |
| **Xiao 12** | 2015 | - | + | - | - | - | + |

^a^ + well described +/- no structured clinical interview performed for controls –no clear definition of included study participants
^b^ + well described methods – methods not well described

^c^ + analysis blinded for outcome assessment – blinding not mentioned
^d^ + baseline characteristics comparable +/- doubtful - systematically different baseline characteristics
^e^ + correction for multipe confounding factors +/- correction for age and gender – no correction for confounders
^f^ + complete information +/- information is limited –important information is missing

**Supplementary Table 3 Confounding factors**

| **Study** | **Age** | **Gender** | **Ethnicity** | **Psychotropic medication** | **Disease state** | **Socioeconomic factors** | **Lifestyle factors** | **Household factors** |
| --- | --- | --- | --- | --- | --- | --- | --- | --- |
| **Avramopoulos ^1^** | R+, C | R+, C | R+, M | NR | NR | NR | NR | NR |
| **Chen ^63^** | R+, C | R+, C | R- | R- | NR | NR | NR | NR |
| **Dickerson ^2^** | R+, M | R+, M | R+, M | R+, M | R+, M | R+, M | NR | R-, M |
| **Gerber ^3^** | R-, M | R+, M | R- | R+ | R+ | M | NR | NR |
| **Hamdani ^4^** | R+ , C | R+, C | R+, C | R+ | R+ | R+, C | R+ | R+ |
| **Prossin ^5^** | R+, C | R+, C | R+, C | R+, M | R+, M | NR | R+, C | NR |
| **Rizzo ^6^** | R+, M, C | R+, M | R-, M | R+, M | R+ | NR | R+, C | NR |
| **Snijders** | R+, C | R+, C | R+, M | R+ | R+ | NR | NR | NR |
| **Tanaka ^7^** | R+, M, C | R+, M | R+, M | R+ | R- | R+, M | R+, M, C | NR |
| **Tedla ^8^** | R-, C | R-, C | R- | R- | R- | NR | NR | M |
| **Abdollahian ^9^** | R+, M | R+, M | R- | NR | NR | R+, M | R+ | R+ |
| **Hinze-Selch ^10^** | R+, M | R+, M | R- | R- | R- | NR | R+, M | R+, M |
| **Khadamavetan ^11^** | R+, M, C | R+, M, C | R+ | NR | NR | R+ | R+ | R+, M |
| **Xiao ^12^** | NR, M | NR, M | R- | NR | NR | NR, M | NR, M | NR, M |

NR = not reported, R - = minimally reported, R + = in detail reported, M = matched or checked if factors have effect on the outcome C = corrected for/in statistical analysis. Socioeconomic factors includes educational level, occupational level, socioeconomic status and profession. Lifestyle factors include smoking, physical activity/condition, body mass index, risky eating habits (raw or uncooked meat, unpasteurized diary products, contaminated water, improper washed food, contact with soil). Household factors include marital status, residence, household with felines.

**Supplementary Table 4 Studies excluded after full-text review**

|  | **Study** | **Reasons** |
| --- | --- | --- |
| **1** | **Allen et al. 1987** ^48^ | No original data |
| **2** | **Alvarado-Esquivel et al. 2011** ^13^ | Sample size < 10 |
| **3** | **Amsterdam et al. 1993** ^14^ | In extractable data, contact information authors not available |
| **4** | **Arling et al. 2009** ^15^ | In extractable data, no response to request for further details |
| **5** | **Brown et al. 2005** ^49^ | No peer-reviewed data, maternal IgG measured |
| **6** | **Burgdorf et al. 2019** ^64^ | Study participants did not meet inclusion criteria |
| **7** | **Cooke et al. 1988** ^50^ | Study participants did not meet inclusion criteria |
| **8** | **Cooke et al. 1989** ^51^ | No original data |
| **9** | **Cooke et al. 1991** ^16^ | No original data |
| **10** | **Coryell et al. 2016** ^17^ | Sample size < 10 |
| **11** | **DeLisi et al. 1986** ^52^ | No original data |
| **12** | **DeLisi et al. 1986** ^53^ | Study participants did not meet inclusion criteria |
| **13** | **DeLisi et al. 1987** ^54^ | Study participants did not meet inclusion criteria |
| **14** | **Dickerson et al. 2006** ^18^ | Possible overlap samples Dickerson et al. 2014, no response by author |
| **15** | **Dickerson et al. 2013** ^19^ | Inextractable data, no response to request for further details |
| **16** | **Dickerson et al. 2014** ^20^ | Inextractable data, no response to request for further details |
| **17** | **Dickerson et al. 2014** ^21^ | Possible overlap samples Dickerson et al. 2014, no response by author |
| **18** | **Dickerson et al. 2015** ^22^ | Inextractable data, no response to request for further details |
| **19** | **Dickerson et al. 2016** ^23^ | Inextractable data, no response to request for further details |
| **20** | **Dickerson et al. 2017** ^24^ | Inextractable data, no response to request for further details |
| **21** | **Dickerson et al. 2018**^55^ | Study participants did not meet inclusion criteria |
| **22** | **Fond et al. 2015** ^25^ | Study participants did not meet inclusion criteria |
| **23** | **Freedman et al. 2016** ^26^ | Maternal IgG measured |
| **24** | **Gale et al. 2018** ^27^ | Study participants did not meet inclusion criteria |
| **25** | **Garcia and Permodo. 1980** ^28^ | No IgG determined |
| **26** | **Halonen et al. 1974** ^29^ | Study participants did not meet inclusion criteria |
| **27** | **Hamdani et al. 2013** ^30^ | Overlap sample of Hamdani et al. 2017, confirmed by author |
| **28** | **Hamdani et al. 2015** ^22^ | Overlap sample Hamdani et al. 2017, confirmed by author |
| **29** | **Hamdani et al. 2018 ^65^** | Overlap sample of Hamdani et al. 2017, confirmed by author |
| **30** | **Hammer et al. unpublished** | No peer-reviewed data |
| **31** | **Houenou et al. 2014** ^31^ | Overlap sample Hamdani et al. 2017, confirmed by author |
| **32** | **Jonker et al. 2017** ^32^ | Study participants did not meet inclusion criteria |
| **33** | **Keller et al. 1990** ^56^ | No full tekst available |
| **34** | **King et al. 1985** ^33^ | Inextractable data, contact information authors not available |
| **35** | **King et al. 1989** ^34^ | No original data |
| **36** | **Lycke et al. 1974** ^35^ | No IgG determined |
| **37** | **Miller et al. 1986** ^57^ | No full tekst available |
| **38** | **Mirza et al. 2012** ^36^ | No IgG determined |
| **39** | **Mortensen et al. 2007** ^37^ | Maternal IgG measured |
| **40** | **Mortensen et al. 2011** ^38^ | Maternal IgG measured |
| **41** | **Nascimento et al. 2012** ^39^ | In extractable data, no response to request for further details |
| **42** | **Olivieira et al. 2016** ^40^ | Overlap sample of Hamdani et al. 2017, confirmed by author |
| **43** | **Pearce et al. 2012** ^41^ | Study participants did not meet inclusion criteria |
| **44** | **Reed et al. 1991** ^58^ | No original data |
| **45** | **Rimon et al. 1969** ^59^ | No full tekst available |
| **46** | **Severance et al. 2014** ^42^ | In extractable data, no response to request for further details |
| **47** | **Simanek et al. 2018** ^43^ | In extractable data, no response to request for further details |
| **48** | **Stich et al. 2015** ^44^ | Study participants did not meet inclusion criteria |
| **49** | **The lancet infectious diseases 2012** ^60^ | No original data |
| **50** | **Torrey et al. unpublished** | No peer-reviewed data |
| **51** | **Van hiele et al. 1988** ^61^ | No original data |
| **52** | **Wang et al. 2006** ^45^ | In extractable data, no response to request for further details |
| **53** | **Yonghua et al. 2014** ^61^ | No peer-reviewed data |
| **54** | **Zhang et al. 2012** ^46^ | In extractable data, no response to request for further details |

**Supplementary Table 5 Overview studies assessing titer levels in bipolar patients and healthy controls**

| **Pathogen** | **Author** | **Year** | **Patients** | **Controls** | **Patiënt IgG titer** | **Controls IgG titer** | **Significant** |
| --- | --- | --- | --- | --- | --- | --- | --- |
| **CMV** | *Avramopoulos* ^1^ | 2015 | 489 | 362 | na | na | N |
| **CMV** | *Dickerson* ^20^ | 2014 | 273 | 314 | na | na | N |
| **CMV** | *Houenou* ^31^ | 2014 | 118 | 102 | 2.63 (2.0) | 2.98 (2.0) | N |
| **CMV** | *Prossin* ^5^ | 2015 | 139 | 99 | 3.0 (2.7) | 2.0 (2.0) | Y |
| **CMV** | *Rizzo* ^6^ | 2013 | 22 | 17 | 3.18 (0.98) | 2.25 (0.61) | Y |
| **CMV** | *Snijders* | 2018 | 760 | 132 | 47.13 (27.13)^a^ | 51.83 (24.63)^a^ | N |
| **CMV** | *Tanaka* ^7^ | 2017 | 32 | 32 | 4.765 (2.84) | 4.031 (2.56) | N |
| **CMV** | *Tedla* ^8^ | 2011 | 199 | 80 | na | na | Y |
| **Herpes viruses'** | *Dickerson* ^47^ | 2015 | 229 | 280 | na | na | N |
| **HSV-1** | *Amsterdam* ^14^ | 1993 | 49 | 30 | 451 (387)^b^ | F: 381 (32), M: (326)^b^ | N |
| **HSV-1** | *Avramopoulos ^1^* | 2015 | 489 | 362 | na | na | N |
| **HSV-1** | *Prossin* ^5^ | 2015 | 139 | 99 | na | na | N |
| **HSV-1** | *Snijders* | 2018 | 760 | 132 | 34.42 (27.61)^a^ | 30.46 (33.48)^a^ | N |
| **HSV-1** | *Tanaka* ^7^ | 2017 | 32 | 32 | 2.047 (1.357) | 1.524 (1.243) | N |
| **HSV-1** | *Tedla* ^8^ | 2011 | 199 | 80 | na | na | N |
| **HSV-2** | *Amsterdam* ^14^ | 1993 | 49 | 30 | 309 (268)^b^ | F: 284 (252) , M: 293 (156)^b^ | N |
| **HSV-2** | *Prossin* ^5^ | 2015 | 139 | 99 | na | na | N |
| **HSV-2** | *Snijders* | 2018 | 760 | 132 | 25.55 (26.68)^a^ | 229.61 (190.59)^a^ | N |
| **HSV-2** | *Tedla* ^8^ | 2011 | 199 | 80 | na | na | N |
| **Toxoplasma** | *Avramopoulos* ^1^ | 2015 | 489 | 362 | na | na | N |
| **Toxoplasma** | *Dickerson* ^47^ | 2015 | 229 | 280 | na | na | N |
| **Toxoplasma** | *Hamdani* ^30^ | 2013 | 110 | 106 | 3.06 (1.7) | 2.07 (1.9) | Y |
| **Toxoplasma** | *Snijders* | 2018 | 760 | 132 | 225.74 (191.17)^a^ | 229.61 (190.59)^a^ | N |
| **Toxoplasmose** | *Tanaka* ^7^ | 2017 | 32 | 32 | 0.710 (0.224) | 0.786 (0.857) | N |
| **Toxoplasmose** | *Tedla* ^8^ | 2011 | 199 | 80 | na | na | N |
| **EBV** | *Snijders* | 2018 | 760 | 132 | 16.31 (5.23)^a^ | 17.39 (5.85)^a^ | N |

**Abbrevations**. F: female. M: male, Y: Yes, N: No.^a^ Titer levels of seropositive cases ^b^ Log-transformed values are given.

**References:**

1 Avramopoulos D, Pearce BD, McGrath J, Wolyniec P, Wang R, Eckart N *et al.* Infection and inflammation in schizophrenia and bipolar disorder: A genome wide study for interactions with genetic variation. *PLoS One* 2015; **10**. doi:10.1371/journal.pone.0116696.

2 Dickerson FB, Boronow JJ, Stallings C, Origoni AE, Cole S, Krivogorsky B *et al.* Infection with herpes simplex virus type 1 is associated with cognitive deficits in bipolar disorder. *Biol Psychiatry* 2004; **55**: 588–593.

3 Gerber SI, Krienke UJ, Biedermann NC, Grunze H, Yolken RH, Dittmann S *et al.* Impaired functioning in euthymic patients with bipolar disorder - HSV-1 as a predictor. *Prog Neuro-Psychopharmacology Biol Psychiatry* 2012; **36**: 110–116.

4 Hamdani N, Daban-Huard C, Godin O, Laouamri H, Jamain S, Attiba D *et al.* Effects of cumulative Herpesviridae and Toxoplasma gondii infections on cognitive function in healthy, bipolar, and schizophrenia subjects. *J Clin Psychiatry* 2017; **78**: e18–e27.

5 Prossin AR, Yolken RH, Kamali M, Heitzeg MM, Kaplow JB, Coryell WH *et al.* Cytomegalovirus antibody elevation in bipolar disorder: Relation to elevated mood states. *Neural Plast* 2015; **2015**. doi:10.1155/2015/939780.

6 Rizzo LB, Do Prado CH, Grassi-Oliveira R, Wieck A, Correa BL, Teixeira AL *et al.* Immunosenescence is associated with human cytomegalovirus and shortened telomeres in type I bipolar disorder. *Bipolar Disord* 2013; **15**: 832–838.

7 Tanaka T, Matsuda T, Hayes LN, Yang S, Rodriguez K, Severance EG *et al.* Infection and inflammation in schizophrenia and bipolar disorder. *Neurosci Res* 2016; **115**: 59–63.

8 Tedla Y, Shibre T, Ali O, Tadele G, Woldeamanuel Y, Asrat D *et al.* Serum antibodies to Toxoplasma gondii and Herpesvidae family viruses in individuals with schizophrenia and bipolar disorder: a case-control study. *Ethiop Med J* 2011; **49**: 211–20.

9 Abdollahian E, Shafiei R, Mokhber N, Kalantar K, Fata A. Seroepidemiological study of Toxoplasma gondii infection among psychiatric patients in Mashhad, Northeast of Iran. *Iran J Parasitol* 2017; **12**: 117–122.

10 Hinze-Selch D, Däubener W, Erdag S, Wilms S. The diagnosis of a personality disorder increases the likelihood for seropositivity to Toxoplasma gondii in psychiatric patients. *Folia Parasitol (Praha)* 2010; **57**: 129–135.

11 Khademvatan S, Khajeddin N, Izadi S, Saki J. Study of Toxoplasma gondii infection in patients with bipolar disorder. *J. Med Sci*. 2013; **13**:215-220.

12 Xiao Y, Yin J, Jiang N, Xiang M, Hao L, Lu H *et al.* Seroepidemiology of human Toxoplasma gondii infection in China. *BMC Infect Dis* 2010; **10**: 0–4.

13 Alvarado-Esquivel C, Sifuentes-Álvarez A, Narro-Duarte SG, Estrada-Martínez S, Díaz-García JH, Liesenfeld O *et al.* Seroepidemiology of Toxoplasma gondii infection in pregnant women in a public hospital in northern Mexico. *BMC Infect Dis* 2006; **6**: 1–7.

14 Amsterdam JD, Hernz WJ. Serum antibodies to herpes simplex virus types I and II in depressed patients. *Biol Psychiatry* 1993; **34**: 417–420.

15 Arling TA, Yolken RH, Lapidus M, Langenberg P, Dickerson FB, Zimmerman SA *et al.* Toxoplasma gondii antibody titers and history of suicide attempts in patients with recurrent mood disorders. *J Nerv Ment Dis* 2009; **197**: 905–908.

16 Cooke RG, Warsh JJ, Hasey GM, McLaughlin BJM, Jorna T. Epstein-barr virus antibodies and severity of depression. *Biol Psychiatry* 1991; **29**. doi:10.1016/0006-3223(91)90102-R.

17 Coryell W, Yolken R, Butcher B, Burns T, Dindo L, Schlechte J *et al.* Toxoplasmosis Titers and past Suicide Attempts Among Older Adolescents Initiating SSRI Treatment. *Arch Suicide Res* 2016; **20**: 605–613.

18 Dickerson FB, Boronow JJ, Stallings C, Origoni AE, Cole S, Leister F *et al.* The catechol O-methyltransferase Val158Met polymorphism and herpes simplex virus type 1 infection are risk factors for cognitive impairment in bipolar disorder: Additive gene-environmental effects in a complex human psychiatric disorder. *Bipolar Disord* 2006; **8**: 124–132.

19 Dickerson F, Stallings C, Origoni A, Vaughan C, Katsafanas E, Khushalani S *et al.* A Combined Marker of Inflammation in Individuals with Mania. *PLoS One* 2013; **8**: 1–6.

20 Dickerson F, Stallings C, Origoni A, Katsafanas E, Schweinfurth L, Savage C *et al.* Antibodies to Toxoplasma gondii and cognitive functioning in schizophrenia, bipolar disorder, and nonpsychiatric controls. *J Nerv Ment Dis* 2014; **202**: 589–593.

21 Dickerson F, Stallings C, Origoni A, Vaughan C, Katsafanas E, Khushalani S *et al.* Antibodies to Toxoplasma gondii in individuals with mania. *Bipolar Disord* 2014; **16**: 129–136.

22 Hamdani N, Daban-Huard C, Lajnef M, Gadel R, Le Corvoisier P, Delavest M *et al.* Cognitive deterioration among bipolar disorder patients infected by Toxoplasma gondii is correlated to interleukin 6 levels. *J Affect Disord* 2015; **179**: 161–166.

23 Dickerson F, Adamos MB, Katsafanas E, Khushalani S, Origoni A, Savage CLG *et al.* The association among smoking, HSV-1 exposure, and cognitive functioning in schizophrenia, bipolar disorder, and non-psychiatric controls. *Schizophr Res* 2016; **176**: 566–571.

24 Dickerson F, Severance E, Yolken R. The microbiome, immunity, and schizophrenia and bipolar disorder. *Brain Behav Immun* 2017; **62**: 46–52.

25 Fond G, Sutterland A. Toxoplasma gondii: Potential therapeutic applications in psychiatry . *Inf Psychiatr* 2016; **92**: 809–814.

26 Freedman D, Bao Y, Shen L, Schaefer CA, Brown AS. Maternal T. gondii, offspring bipolar disorder and neurocognition. *Psychiatry Res* 2016; **243**: 382–389.

27 Gale SD, Berrett AN, Erickson LD, Brown BL, Hedges DW. Association between virus exposure and depression in US adults. *Psychiatry Res* 2018; **261**: 73–79.

28 Delgado Garcia G, Rodriguez Perdomo E. [Reactivity of toxoplasmin intradermal test in neurotic and manic-depressive patients]. *Rev Cubana Med Trop* 1980; **32**: 35–39.

29 Halonen PE, Rimon R, Arohonka K, Jantti V. Antibody levels to herpes simplex type I, measles and rubella viruses in psychiatric patients. *Br J Psychiatry* 1974; **125**: 461–465.

30 Hamdani N, Daban-Huard C, Lajnef M, Richard JR, Delavest M, Godin O *et al.* Relationship between Toxoplasma gondii infection and bipolar disorder in a French sample. *J Affect Disord* 2013; **148**: 444–448.

31 Houenou J, d’Albis MA, Daban C, Hamdani N, Delavest M, Lepine JP *et al.* Cytomegalovirus seropositivity and serointensity are associated with hippocampal volume and verbal memory in schizophrenia and bipolar disorder. *Prog Neuro-Psychopharmacology Biol Psychiatry* 2014; **48**: 142–148.

32 Jonker I, Rosmalen JGM, Schoevers RA. Childhood life events, immune activation and the development of mood and anxiety disorders: The TRAILS study. *Transl Psychiatry* 2017; **7**: 1–9.

33 King DJ, Cooper SJ, Earle JAP, Martin SJ, McFerran N V., Rima BK *et al.* A survey of serum antibodies to eight common viruses in psychiatric patients. *Br J Psychiatry* 1985; **147**: 137–144.

34 King DJ, Cooper SJ. Viruses, immunity and mental disorder. Br. J. Psychiatry. 1989; **154**: 1–7.

35 Lycke E, Norrby R, Roos BE. A Serological Study on Mentally Ill Patients With Particular Reference to the Prevalence of Herpes Virus Infections. *Br J Psychiatry* 1974; **124**: 273–279.

36 Mirza RA, Eick-Cost A, Otto JL, R.A. M, A. E-C, J.L. O. The risk of mental health disorders among U.S. military personnel infected with human immunodeficiency virus, active component, U.S. Armed Forces, 2000-2011. *MSMR* 2012; **19**: 10–13.

37 Mortensen PB, Nørgaard-Pedersen B, Waltoft BL, Sørensen TL, Hougaard D, Torrey EF *et al.* Toxoplasma gondii as a Risk Factor for Early-Onset Schizophrenia: Analysis of Filter Paper Blood Samples Obtained at Birth. *Biol Psychiatry* 2007; **61**: 688–693.

38 Mortensen PB, Pedersen CB, Mcgrath JJ, Hougaard DM, Nørgaard-Petersen B, Mors O *et al.* Neonatal antibodies to infectious agents and risk of bipolar disorder: A population-based case-control study. *Bipolar Disord* 2011; **13**: 624–629.

39 Nascimento FS, de Rosalmeida Dantas C, Netto MP, Mella LFB, Suzuki LA, Banzato CEM *et al.* Prevalence of antibodies to Toxoplasma gondii in patients with schizophrenia and mood disorders. *Schizophr Res* 2012; **142**: 244–245.

40 Oliveira J, Kazma R, Le Floch E, Bennabi M, Hamdani N, Bengoufa D *et al.* Toxoplasma gondii exposure may modulate the influence of TLR2 genetic variation on bipolar disorder: a gene–environment interaction study. *Int J Bipolar Disord* 2016; **4**: 11.

41 Pearce BD, Kruszon-Moran D, Jones JL. The relationship between Toxoplasma Gondii infection and mood disorders in the third national health and nutrition survey. Biol. Psychiatry. 2012; **72**: 290–295.

42 Severance EG, Gressitt KL, Stallings CR, Origoni AE, Khushalani S, Leweke FM *et al.* Discordant patterns of bacterial translocation markers and implications for innate immune imbalances in schizophrenia. *Schizophr Res* 2013; **148**: 130–137.

43 Simanek AM, Parry A, Dowd JB. Differences in the association between persistent pathogens and mood disorders among young- to middle-aged women and men in the U.S. Brain. Behav. Immun. 2017. doi:10.1016/j.bbi.2017.09.017.

44 Stich O, Andres TA, Gross CM, Gerber SI, Rauer S, Langosch JM. An observational study of inflammation in the central nervous system in patients with bipolar disorder. *Bipolar Disord* 2015; **17**: 291–302.

45 Wang HL, Wang GH, Li QY, Shu C, Jiang MS, Guo Y. Prevalence of Toxoplasma infection in first-episode schizophrenia and comparison between Toxoplasma-seropositive and Toxoplasma-seronegative schizophrenia. *Acta Psychiatr Scand* 2006; **114**: 40–48.

46 Zhang Y, Träskman-Bendz L, Janelidze S, Langenberg P, Saleh A, Constantine N *et al.* Toxoplasma gondii immunoglobulin G antibodies and nonfatal suicidal self-directed violence. *J Clin Psychiatry* 2012; **73**: 1069–1076.

47 Dickerson F, Katsafanas E, Schweinfurth LAB, Savage CLG, Stallings C, Origoni A *et al.* Immune alterations in acute bipolar depression. *Acta Psychiatr Scand* 2015; **132**: 204–210.

48 Allen AD, Fudenberg HH, Allen RE. Affective disorder and viral infections. *Arch Gen Psychiatry*. 1987; **44:**760.

49 Brown AS, Montoya JG, Bao Y, Shen L, Schaefer CA. Specificity of maternal Toxoplasma gondii to risk of schizophrenia in offspring. *Schizophr Bull* 2013:39:S59.

50 Cooke RG, Langlet F, McLaughlin BJ. Age-specific prevalence of Epstein-Barr virus antibodies in adult patients with affective disorders. *J Clin Psychiatry*. 1988;**49:**361-3.

51 Cooke RG, Warsh JJ, Hasey GM. Epstein-Barr virus as a cause of autoimmune disease and other medical morbidity

in patients with affective disorders. *Med Hypotheses*. 1989; **29:**177-85.

52 DeLisi. Immunological disturbances in psychiatric patients. *Arch Gen Psychiatry*. 1986;43:189-91.

53 DeLisi LE, Nurnberger JS, Goldin LR, Simmons-Alling S, Gershon ES. Epstein-Barr virus and depression. *Arch Gen Psychiatry*. 1986; **43**:815-6.

54 DeLisi. Epstein-Barr virus and depression. *Am J Psychiatry*. 1987; **144**:1374-5.

55 [Dickerson F](https://www.ncbi.nlm.nih.gov/pubmed/?term=Dickerson%20F%5BAuthor%5D&cauthor=true&cauthor_uid=29474231), [Origoni A](https://www.ncbi.nlm.nih.gov/pubmed/?term=Origoni%20A%5BAuthor%5D&cauthor=true&cauthor_uid=29474231), [Schweinfurth LAB](https://www.ncbi.nlm.nih.gov/pubmed/?term=Schweinfurth%20LAB%5BAuthor%5D&cauthor=true&cauthor_uid=29474231), [Stallings C](https://www.ncbi.nlm.nih.gov/pubmed/?term=Stallings%20C%5BAuthor%5D&cauthor=true&cauthor_uid=29474231), [Savage CLG](https://www.ncbi.nlm.nih.gov/pubmed/?term=Savage%20CLG%5BAuthor%5D&cauthor=true&cauthor_uid=29474231), [Sweeney K](https://www.ncbi.nlm.nih.gov/pubmed/?term=Sweeney%20K%5BAuthor%5D&cauthor=true&cauthor_uid=29474231) *et al.* Clinical and Serological Predictors of Suicide in Schizophrenia and Major Mood Disorders. [*J Nerv Ment Dis.*](https://www.ncbi.nlm.nih.gov/pubmed/29474231) 2018; **206**:173-178.

56 Keller SE, Schleifer SJ, Bartlett JA. Depression, altered immunity, and health: clinical implications for psychoimmunologic processes. *Res Publ Assoc Res Nerv Ment Dis*. 1990;68:179-82.

57 Miller AH, Silberstein C, Asnis GM, Munk G, Rubinson E, Spigland I, Norin A. Epstein-Barr virus infection and depression. *J Clin Psychiatry*. 1986; **47**:529-30.

58 Reed SM, Glick JW. Fluoxetine and reactivation of the herpes simplex virus. Am J Psychiatry. 1991 **148:**949-50.

59 Rimon R, Halonen P. Herpes simplex virus infection and depressive illness. *Dis Nerv Syst*. 1969 **30**:338-40.

60 The Lancet Infectious Diseases. Toxoplasma gondii: an unknown quantity. *Lancet Infect Dis*. 2012 **12**:737.

61 van Hiele LJ. ['It's bound to be a virus...']. Ned Tijdschr Geneeskd. 1988 ;**132**:642.

62 Yonghua Z, Yue C, Lei Z. Analysis of probable correlation between Toxoplasma gondii and schizophrenia: a sero-epidemiological longitudinal

investigation from 2002 to 2007 in Suzhou and Wuxi regions. Jiangsu, China. *Trop Med Int Health* 2011;**16**:97-384.

63 Chen X, Chen B, Hou X, Zheng C, Yang X, Ke J *et al.* Association between Toxoplasma gondii infection and psychiatric disorders in Zhejiang, Southeastern China. *Acta Trop* 2019; **192**: 82–86.
